# Supplementary material for: Thirteen dubious ways to detect conserved structural RNAs
Source: IUBMB Life. Author manuscript; Available in PMC 2024 Jul 10. (PMC11234323; doi:10.1002/iub.2694)
Supplement: supplementary_material [file NIHMS1907110-supplement-supplementary_material.gz › supplemental_material/Table1/CRS.structural.tar.gz!/selected/selected_noRfam/noNUMTs/cmf.M0950988/MyM0950988/R-scape_nhmmer/cmf.M0950988_ext_liftover.hg38.R2R.sto.pdf]

cmf.M0950988_ext_liftover.hg38

5´-G G U U C U A A U C A G A U G C U G G C A G C U G A G G A C U C C C C A G G A A U G Y G C C G G G A U G U U U U U G C U G C C U G G G C U Y G G U A A C U C C C C C U C A A C G U G U C U G C Y G A G U C U U C C A A A C U U A A C U G U U U A U U A A C U C R ● G C Y R ● ● G ○
